# Curcumin-carrying nanoparticles prevent ischemia-reperfusion injury in human renal cells

**DOI:** 10.18632/oncotarget.13626

**Published:** 2016-11-25

**Authors:** Yong Xu, Ning Hu, Wei Jiang, Hong-Fang Yuan, Dong-Hui Zheng

**Affiliations:** ^1^ Department of Nephrology, The Affiliated Huai'an Hospital of Xuzhou Medical University and The Second People's Hospital of Huai'an, Huai'an 223002, China; ^2^ Department of Nephrology, The First People's Hospital of Jingmen, Jingmen, Hubei 448000, China; ^3^ Family Planning Research Institute, Tongji Medical College, Huazhong University of Science and Technology, Wuhan 430030, China

**Keywords:** Curcumin, nanoparticles, ischemia-reperfusion injury

## Abstract

Renal ischemia-reperfusion injury (IRI) is a major complication in clinical practice. However, despite its frequency, effective preventive/treatment strategies for this condition are scarce. Curcumin possesses antioxidant properties and is a promising potential protective agent against renal IRI, but its poor water solubility restricts its application. In this study, we constructed curcumin-carrying distearoylphosphatidylethanolamine-polyethylene glycol nanoparticles (Cur-NPs), and their effect on HK-2 cells exposed to IRI was examined in vitro. Curcumin encapsulated in NPs demonstrated improved water solubility and slowed release. Compared with the IRI and Curcumin groups, Cur-NP groups displayed significantly improved cell viability, downregulated protein expression levels of caspase-3 and Bax, upregulated expression of Bcl-2 protein, increased antioxidant superoxide dismutase level, and reduced apoptotic rate, reactive oxygen species level, and malondialdehyde content. Results clearly showed that Cur-NPs demonstrated good water solubility and slow release, as well as exerted protective effects against oxidative stress in cultured HK-2 cells exposed to IRI.

## INTRODUCTION

Ischemia-reperfusion injury (IRI) is the damage caused by unavailability of blood supply in an organ until blood flow and oxygenation are restored [1]. Given the specificity in structure and function of the kidney, renal IRI commonly occurs and is associated with many complex clinical conditions, such as hemorrhagic shock [2], renal transplantation [3], and acute renal failure [4]. Pathogenic studies have shown that apoptosis plays a vital role in renal IRI and is reduced by an imbalance in scavenging and generation of reactive oxygen species (ROS) [5, 6], but the related mechanism is poorly understood. To our knowledge, effective preventive/treatment strategies for this condition are scarce.

Curcumin, an active component extracted from the roots and stems of *Curcuma* Species, such as turmeric, radix curcumae, and rhizoma curcumae, demonstrates antioxidant properties [7, 8]. A study has further indicated that curcumin ameliorates tubular necrosis by significantly reducing the formation of noxious oxidants [9]. Although Curcumin is easily metabolized, its application has been restricted because of its poor water solubility and thus is difficultly absorbed by the body. Therefore, new dosage forms of Curcumin must be developed to increase its solubility, to enhance its pharmacological effects, and to facilitate proper route of administration.

Two comprehensive reviews have recently reported that drug delivery using nanoparticles (NPs) offers numerous advantages, such as targeted delivery, slowed release, high stability, and relatively low toxicity [10, 11]. NPs prepared from distearoylphosphatidylethanolamine-polyethylene glycol (DSPE-PEG) display bidirectional solubility in water and in fat. PEG demonstrates good water solubility and low immunogenicity and thus can be excreted from the kidney without need for any structural changes [12]. DSPE displays good drug encapsulation efficiency and therefore can be used as core [13]. The bidirectional solubility of DSPE-PEG increases the encapsulation efficiency and water solubility of fat-soluble drugs.

Given the advantages of nanocarriers and the pharmaceutical features of Curcumin, Curcumin-carrying long-circulating DSPE-PEG NPs were constructed in this study. The effect of these NPs on HK-2 cells exposed to IRI was examined in vitro, and the mechanism of such effect was preliminarily investigated.

## RESULTS

### Solubility of Curcumin and Cur-NPs

**Figure 1:**
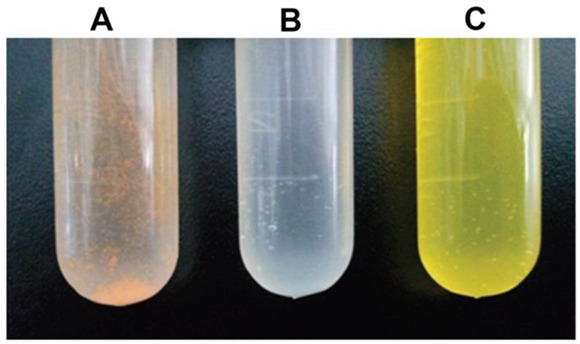
The solution of Cur-NPs in test tube **A**. Non-encapsulated curcumin **B**. Blank nanoparticles **C**. Cur-NPs.

Precipitation of Curcumin in water (Figure 1A) demonstrated that Curcumin is poorly water soluble. The water solution of blank NPs is light blue and transparent, whereas that of Cur-NPs is yellow and transparent, suggesting that encapsulation in DSPE-PEG NPs enhanced the water solubility of Curcumin (Figure 1C).

### Size of DSPE-PEG NPs

**Figure 2:**
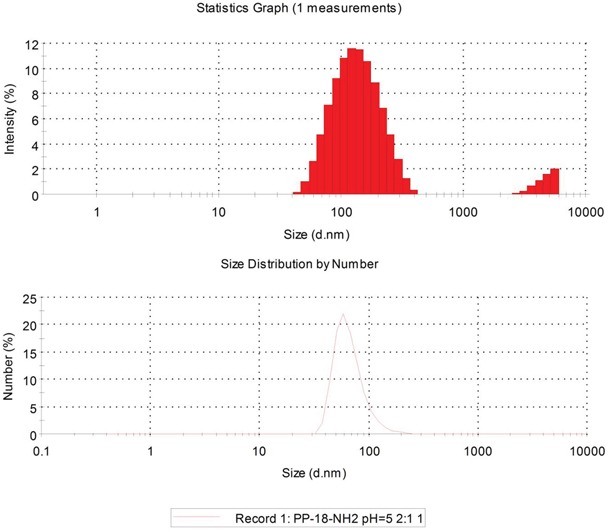
Size and Zeta potential of DSPE-PEG nanoparticles

The mean size of Cur-NPs was 81.93 ± 0.5 nm. The DSPE-PEG NPs showed a narrow size distribution as determined using a particle size analyzer (Figure 2). Additionally, Lestari [14] reported that a nanosystem can maintain long-term stability when its zeta potential is −11.1 mV.

### In vitro investigation on drug release

**Figure 3:**
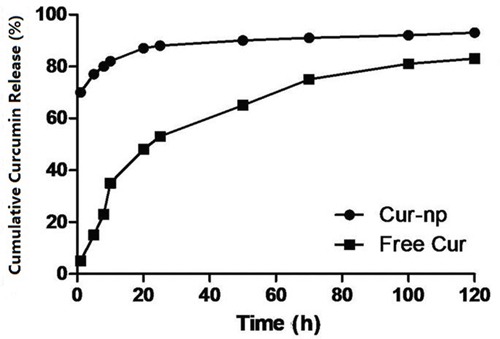
Release curve of Curcumin drug-loading nanoparticles and bare curcumin medicine in vitro

Figure 3 shows that the release rate of Cur-NPs was significantly slower than that of non-encapsulated Curcumin. The percentages of released non-encapsulated Curcumin and Cur-NPs from a dialysis bag for the first 5 h were approximately 80% and approximately 30%, respectively. After 40 h, the percentage of Cur-NPs released from the dialysis bag was approximately 60%. These results indicated that Cur-NPs can be slowly released, thereby maintaining a constant concentration over a long period.

### Effects of Curcumin and Cur-NPs on IRI-inhibited cell proliferation

**Figure 4:**
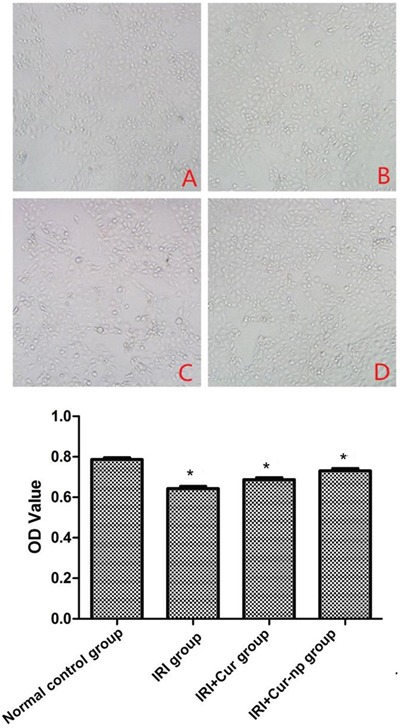
Detection of cell proliferation determined by MTT method

Figure 4 shows that IRI treatment significantly inhibited the proliferation of HK-2 cells; this phenomenon was indicated by the changes in optical density (OD) as revealed by methyl thiazolyl tetrazolium (MTT) colorimetry (*p* < 0.05). By contrast, Curcumin treatment significantly alleviated the inhibitory effects of IRI on cell proliferation (*p* < 0.05), and Cur-NP treatment further alleviated the inhibitory effects of IRI on cell proliferation compared with Curcumin treatment (*p* < 0.05). These results indicated that, compared with Curcumin, Cur-NPs exert higher protective effects against IRI injury in HK-2 cells.

### Effects of Curcumin and Cur-NPs on apoptosis of HK-2 cells

Four groups of HK-2 cells were assessed. Data derived from flow cytometry profiles were qualitatively consistent with the results of replicate experiments. Treated cells were cultured for 48 h, harvested, stained with Annexin V-FITC/PI, and then analyzed via flow cytometry.

**Figure 5:**
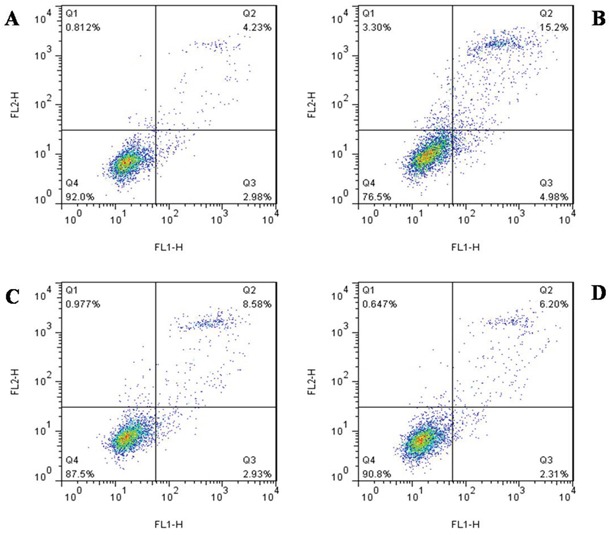
The change of apoptosis detected flow cytometry **A**. Normal control group. **B**. IRI group. **C**. IRI+Cur group. **D**. IRI+Cur-NPs group.

Annexin V was used in flow cytometric analysis to examine the level of apoptosis in HK-2 cells. As shown in Figure 5, the Cur-NP group displayed a reduced number of apoptotic cells (15.20% in the injury group vs. 6.20% in the Cur-NP group). These results demonstrated that the number of early and late apoptotic cells decreased in the Curcumin group (Figure 5). The percentages of cells in the early apoptotic phase were 4.23%, 15.20%, 8.58%, and 6.20% in the control, injury, Curcumin, and Cur-NP groups, respectively, and the corresponding percentages of cells in the late apoptotic phase were 2.98%, 4.98%, 2.93%, and 2.31% (Figure 5). Data suggested that Cur-NPs can protect HK-2 cells and that they can better alleviate apoptosis than Curcumin.

### Determination of intracellular reactive oxygen content by using DCFH-DA fluorescence probe

**Figure 6:**
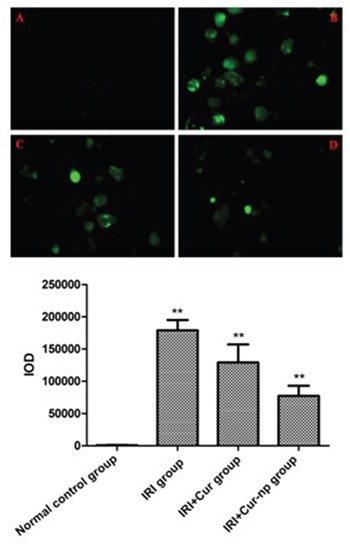
The results of DAPI staining after hypoxia reoxygenation at 24h **A**. Normal control group; **B**. IRI group; **C**. IRI+Cur group; **D**. IRI+Cur-NPs group.

Intracellular ROS level was detected via fluorescent staining. The IR cells clearly showed an enhanced intensity of yellow-green fluorescence, demonstrating that intracellular ROS level increased after IRI; this result indicated that oxidative stress response plays an important role in renal IRI. Fluorescence intensity decreased both in the IRI+Curcumin and IRI+Cur-NP groups (co-cultured with HK-2 cells), although fluorescence intensity was more significantly reduced in the IRI+Cur-NP group than in the IRI+Curcumin group. This result demonstrated that Cur-NPs can effectively prevent and treat renal IRI by inhibiting oxidative stress response (Figure 6).

### Effects of Curcumin and Cur-NP on malondialdehyde (MDA), superoxide dismutase (SOD), Caspase-3, Bax, and Bcl-2 levels following IRI

**Figure 7:**
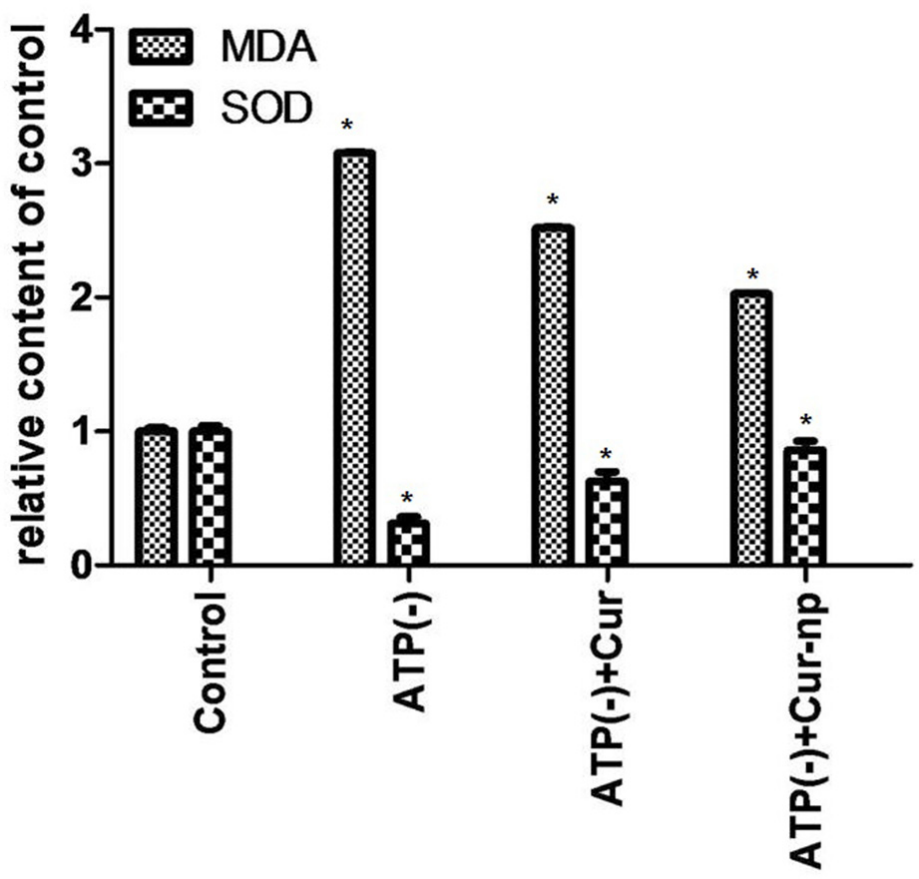
MDA and SOD tests

Intracellular SOD and MDA contents were determined using the bicinchoninic acid method. MDA level decreased after adding Cur-NPs or non-encapsulated Curcumin. Such reduction was significantly greater in the Cur-NP group than in the Curcumin group. The SOD level increased after adding Cur-NPs or non-encapsulated Curcumin, although such an increase was significantly greater in the Cur-NP group than in the Curcumin group. This result indicated that Curcumin-carrying NPs increased the SOD content and reduced the MDA level (Figure 7).

**Figure 8:**
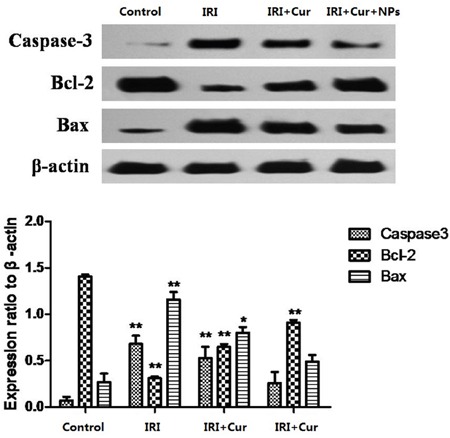
Changes in Expression levels of caspase-3, Bax, and Bcl-2 proteins in each group

Expression levels of Caspase-3, Bax, and Bcl-2 proteins in each group were detected by Western blot assay. The results showed that addition of Cur-NPs or non-encapsulated Curcumin downregulated the protein expression levels of Caspase-3 and Bax but upregulated Bcl-2 expression. Increase in expression levels was significantly higher in the Cur-NP group than in the Curcumin group (Figure 8).

## DISCUSSION

This study successfully constructed Cur-NPs that display good water solubility and pharmacological features. Our experiments demonstrated that Cur-NPs inhibited the apoptotic rate of HK-2 cells exposed to IRI by reducing reactive oxygen level, by downregulating the expression of pro-apoptotic genes (Caspase-3 and Bax), and by upregulating the expression of anti-apoptotic gene (Bcl-2).

Curcumin possesses anti-microbial [15], anti-inflammatory [16], anti-carcinogenic [17], and anti-oxidant [18] properties. In spite of these properties, the wide use of Curcumin has been limited mainly by its poor water solubility and consequently its poor bioavailability. Intravenous administration of Curcumin, which is a small fat-soluble molecule, is inconvenient and unsafe; moreover, the disadvantages of Curcumin includes its low bioavailability, short half-life, and difficulty in reaching and maintaining therapeutic concentration after entering the body [19]. Large-dose administration not only fails to significantly increase the in vivo concentration of Curcumin but also induces oxidative stress, generating concentration-dependent toxicity [20]. Thus, developing innovative form of Curcumin to increase its bioavailability and reduce its toxic side effects is extremely necessary. Sasaki [21] described an innovative preparation of Curcumin, in which Curcumin demonstrates improved oral bioavailability and displays the characteristics of nanomaterials. Given the safety, high stability under UV light and high temperature, and excellent water solubility of nanomaterials, they have become increasingly used as effective components in preparing new health care products (beverages, food, supplements, and drugs) [22]. Our data clearly showed that Cur-NPs displayed obviously improved water solubility compared with non-encapsulated Curcumin; moreover, Cur-NPs are approximately 80 nm in size; thus, they are easily absorbed by the body and that they reach their destination. These results are consistent with those of other studies that have taken advantage of the water solubility of NPs [21, 23]. In addition, investigation on in vitro drug release showed that cumulative release of Cur-NP was significantly higher than that of non-encapsulated Curcumin, indicating that NPs facilitate the release of Curcumin. High cumulative release concentration is obviously essential in increasing the bioavailability of Curcumin. The release behavior may be induced by improved solubility of Curcumin in water, consistent with the findings of Li [24] and Kumar [25].

Although the pathophysiology of IRI is incompletely clear, oxidative stress has been identified as one of the several important mechanisms causing kidney failure [26, 27]. When a kidney experiences ischemia and then reperfusion, excessive ROS generation at the latter phase of ischemia and reperfusion initiates a series of deleterious cellular responses, resulting in inflammation, cell apoptosis, and acute kidney failure [1]. Renal tissues receive abundant blood supply, and the enzymatic and non-enzymatic antioxidant systems are relatively weak [28]. Hence, renal tissues are more sensitive to oxidative stress than the other organs. Rapid elimination of excess ROS during the early stage of ischemia and reperfusion can protect tubular epithelial cells, attenuating the adverse influence of ROS on renal tissues. This study used HK-2 cells to prepare an IRI model. After ischemia-reperfusion (IR), cell viability gradually decreased, whereas the detected ROS level in the cells obviously increased, suggesting that during renal IRI, the number of reactive oxygen molecules and the apoptotic rate significantly increased and that oxidative stress response plays an important role in IRI.

Endogenous substances necessary for ROS elimination include antioxidant enzymes (e.g., SOD, glutathione peroxidase, and catalase) and non-enzymatic antioxidants (e.g., glutathione, uric acid, and vitamins C and E) [29]. Under abnormal states, the capacity to eliminate free radicals from a system is usually impaired. Exogenous antioxidant supplementation is theoretically one of the most effective treatment strategies for IRI [30, 31]. Studies have shown that antioxidants can be exogenously supplemented through overexpression of antioxidant enzymes by using viral vectors or via direct supplementation with antioxidant enzymes and small molecule antioxidants to treat different IRI types [32–35]. However, overexpression of antioxidant enzymes by using viral vectors is a complex technology and involves problems, such as the concerns on immune responses and slow effects. By contrast, antioxidant enzymes and small-molecule antioxidants, such as vitamin C, cannot achieve the treatment goal given their short half-life in vivo. Curcumin, which displays a powerful antioxidant capability, is a promising candidate as treatment for renal IRI.

Caspase-3 is considered the primary terminal restriction enzyme in renal IRI-induced apoptosis of tubular epithelial cells [35, 36]. Moreover, renal tissue apoptosis is closely related to the expression of the regulatory genes Bcl-2 and Bax [37, 38]. Bcl-2 inhibits apoptosis by inhibiting free radical production, intracellular calcium overload, permeability of mitochondrial membrane, and blockage of Cyt-C release and caspase activation. Bax is a pro-apoptotic gene that induces apoptosis by promoting cytokine deficiency. Bcl-2 and Bax may form dimers, the ratio of which determines whether a cell survives or becomes apoptotic. MDA is a main product of lipid peroxidation. MDA reflects the degree of damage in tissues and cells caused by lipid peroxidation [39]. In this study, MDA, SOD, apoptosis, caspase-3, Bax, and Bcl-2 proteins were chosen as detection parameters, and Curcumin and Cur-NPs were used as intervention for HK-2 cells exposed to IRI. Compared with the IRI group, the Curcumin and Cur-NP groups displayed improved cell viability, reduced apoptotic level and MDA content, reduced expression of Caspase-3 protein, downregulated expression of Bax protein, upregulated expression of Bcl-2 protein, and increased level of the antioxidant SOD. These improvements observed in the Cur-NP group were more significant than those in the Curcumin group, indicating that Curcumin can effectively prevent and treat renal IRI by inhibiting oxidative stress response. Furthermore, NPs can significantly promote the inhibitory effect of Curcumin, enhancing the prevention and treatment of renal IRI.

In our study, Cur-NPs demonstrated good water solubility and slowed release, as well as exerted protective effects against oxidative stress in cultured HK-2 cells exposed to renal IRI. Cur-NPs more effectively exerted protective effects than the non-encapsulated Curcumin. Cur-NPs can significantly reduce HK-2 apoptosis and protect the cells by scavenging excessive ROS, by inhibiting the expression levels of Caspase-3 and Bax protein, and by increasing the expression of Bcl-2. Thus, Cur-NPs may be developed as a promising potential protective agent against renal IRI. Further studies on the effect of Cur-NPs must be conducted.

## MATERIALS AND METHODS

### Reagents and instruments

Curcumin (C_21_H_20_O_6_; >98% pure) was purchased from Shaanxi Sciphar Biotechnology Co., Ltd. DSPE and PEG were purchased from Sigma Inc. Apoptosis detection kit (Annexin VFITC/PI), ROS detection kit, protein extraction kit, MDA kit, and SOD kit were all purchased from Nanjing Key Gen Biotech. Co., Ltd.

The following instruments were used: CO_2_ incubator (Thermo311, USA), CO_2_ tri-gas incubator (Thermo3131, USA), microplate reader (ELx800, BioTek, USA), flow cytometer (FACSCalibur, Becton-Dickinson, USA), inverted fluorescence microscope (IX51, OLYMPUS, Japan), Western electrophoresis apparatus (164-5051, Bio-Rad, USA), spectrophotometer (UV-2540, Shimadzu, Japan), and gel imager (Gel Doc XR, Bio-Rad, USA).

### Cell culture

The human renal tubular epithelial cell line HK-2 was provided by Nanjing Key Gen Biotech. Co., Ltd. and cultured in a DMEM/F12 culture solution containing 10% fetal bovine serum (FBS), 100 U/mL penicillin, and 100 mg/L streptomycin. The cells were cultured at 37°C under 5% CO_2_ saturated humidity. Cells in the logarithmic phase were chosen for the experiments.

### Construction of Cur-DSPE-PEG NPs (Cur-NPs)

Cur-NPs were constructed as previously reported. Briefly, an amount of Res and 20 mg of PVP-b-PCL diblock copolymer were dissolved in 0.4 mL of acetone. Under moderate stirring at 25°C, the solution above was added dropwise into 5 mL of distilled water. The resulting solution was filtered through a 0.22 μm filter membrane to remove the unincorporated copolymer aggregates and drugs. The solution was subsequently placed into a dialysis bag (MWCO 12000) and then immersed in redistilled water to thoroughly remove the acetone. Finally, the solution was lyophilized for further use. Empty NPs were prepared in a similar manner but omitting the drug. Green fluorescent coumarin-6 was incorporated into the NPs to visualize the uptake of polymeric micelles by cells. The lyophilized NPs were dissolved in PBS before use.

### In vitro investigation on drug release

Curcumin release was investigated in vitro by using a dialysis bag. Lyophilized Cur-NPs (10 mg) was suspended in 1 mL of physiological saline. Equal amounts of Cur-NPs solution and non-encapsulated Curcumin were placed into a dialysis bag (molecular weight cut-off of 12,000), which was completely immersed in 0.01 mol/L phosphate-buffered saline (PBS, pH = 7.4). The entire release experiment was performed at 37°C. At regular intervals, extracellular fluid was removed and an equal amount of new PBS was added. Curcumin content in the outer liquid was measured at different time points through high performance liquid chromatography.

### Setting of IRI conditions

Cells were washed two to three times with PBS, and complete medium containing 10% FBS was added. The cells were subsequently placed in a hypoxia device (<1% oxygen content). Aeration proceeded for 0.5 h, and then the device was sealed. The cells were continuously cultured for 24 h under ischemia and hypoxia, and then the culture medium was replaced with the same type and volume of medium on which the cells were cultured for another 24 h.

### Examination of cell proliferation using the MTT method

HK-2 cells in the logarithmic phase were diluted to a density of 5 × 10^4^ cells/mL by using DMEM/F12 containing 10% FBS and then inoculated in a 96-well culture plate; each well contains 100 μL of medium. The experiment included a normal control group, IR group, IR+Curcumin (IR-Cur) group (50 μM), and IR+Cur-carrying NP (IR-Cur-NP) group (50 μM). Different drug concentrations were separately added into the wells and then the plate was incubated at 37°C under 5% CO_2_ for 24 h. After incubation, 20 μL of MTT (5 mg/mL) was added into each well, and the plate was continuously incubated at 37°C for another 4 h. The supernatant was discarded and 150 μL of dimethylsulfoxide was added into each well. The plate was mildly shaken in a shaker for 10 min to adequately dissolve the crystals. OD was measured using an automatic microplate reader (wavelength, 490 nm), and cell inhibition rates under different drug concentrations were calculated by the following formula: Cell inhibition rate = (OD value of the control group − OD value of the experimental group/OD value of the control group) × 100%. Five parallel control wells were used in each group.

### Apoptosis detection through flow cytometry

Cells in the logarithmic phase were digested and then inoculated on a six-well plate. Once the cells adhered onto the wall the following day, the corresponding drug-containing culture media were added into the cells of the different groups. At the same time, a negative control group was set. After 24 h of drug action, 0.25% trypsin (EDTA-free) was used to digest the collected cells, which were subsequently washed twice with PBS (centrifuged at 2,000 rpm for 5 min) to collect 5 × 10^5^ cells. A binding buffer (500 μL) was added to suspend the cells. After the cell suspension was mixed thoroughly with 5 μL of Annexin V-FITC, 5 μL of propidium iodide was added and mixed thoroughly into the mixture. The system was allowed to react in the dark for 5–15 min at room temperature. The apoptotic conditions were subsequently examined via flow cytometry.

### Observation of changes in reactive oxygen content in cells through fluorescence microscopy

Cells were washed once with PBS and then centrifuged at 2,000 rpm for 5 min. The cells were subsequently collected, and cell concentration was adjusted to 1 × 10^6^ cells/mL. 2′, 7′-Dichlorofluorescin diacetate (DCFH-DA) was diluted with a serum-free culture solution at 1:1,000 to reach a final concentration of 10 μM. The cells were collected, suspended in prediluted DCFH-DA, and then incubated at 37°C for 20 min. The cell culture was turned upside down every 3–5 min to mix the cells and ensure adequate contact between the probes and the cells. The cells were subsequently washed with a serum-free cell culture solution for three times to completely eliminate the DCFH-DA that did not penetrate into the cells. Fluorescence intensity was determined and photographs were obtained via fluorescence microscopy.

### Determination of SOD and MDA contents

After cessation of drug action on the cells, the culture medium was sucked out. The cells were washed three times with a post-nuclear supernatant, digested with 0.25% trypsin, and then the cell suspension was collected. SOD and MDA contents were determined through spectrophotometry according to the instruction manual.

### Detection of protein expression through western blot assay

After cessation of drug action on the cells, the cells were digested with trypsin and then washed twice with cold PBS. Radioimmunoprecipitation assay lysis buffer (200 μL) was added for complete cell lysis. The mixture was thoroughly mixed and then placed in an ice bath for 30 min, during which the mixture was mixed on a vortex mixer for 10 s every 5 min to achieve complete lysis. The mixture was subsequently centrifuged at 12,000 rpm for 5 min for protein extraction, and protein concentration was determined on a spectrophotometer by using the Coomassie brilliant blue G250 method followed by 12% sodium dodecyl sulfate-polyacrylamide gel electrophoresis electrophoretic separation. The protein was electrotransfered onto a polyvinylidenefluoride membrane, blocked by using 5% skim milk powder for 1 h followed by blocking with a primary antibody (1:500) at 4°C overnight, and then incubated with secondary antibody (horseradish peroxidase-labeled goat anti-rabbit or goat anti-rat antibody 1:1,000) for 2 h. The results were obtained using the chemiluminescence method, and photographs were captured using a gel image analysis system.

### Statistical analyses

Data are presented as mean ± standard error. Comparisons were made by using one-way ANOVA followed by multiple comparison (Fisher's least significant difference test), which is available in the SPSS13.0 statistical software (SPSS, Chicago, Illinois, USA). A *p* value of <0.05 indicated statistical significance.
